# Survival benefit of a low ratio of visceral to subcutaneous adipose tissue depends on LDL clearance versus production in sepsis

**DOI:** 10.1186/s13054-018-1985-1

**Published:** 2018-03-06

**Authors:** Joseph G. H. Lee, Kelly R. Genga, Chawika Pisitsak, John H. Boyd, Alex K. K. Leung, James A. Russell, Keith R. Walley

**Affiliations:** 1 Centre for Heart Lung Innovation, University of British Columbia, Vancouver, BC Canada; 2 Ramathibodi Hospital, Faculty of Medicine, Mahidol University, Bangkok, Thailand

**Keywords:** Sepsis, Visceral abdominal fat, PCSK9 genotype, LDL-cholesterol

## Abstract

**Background:**

Patients with sepsis with a high ratio of visceral adipose tissue (VAT) to subcutaneous adipose tissue (SAT) have increased mortality. Our goal was to investigate the mechanism of this effect, noting that low LDL levels are also associated with increased sepsis mortality. Accordingly we tested for association between VAT/SAT, low-density lipoprotein (LDL) levels, and mortality. Then we examined the effect of statin treatment, which decreases LDL production, and the effect of PCSK9 genotype, which increases LDL clearance.

**Methods:**

We performed retrospective analysis of a cohort of patients with sepsis from a tertiary care adult intensive care unit in Vancouver, Canada, who underwent abdominal computed tomography (CT) (*n* = 75) for clinical reasons. We compared LDL levels in patients with sepsis according to high versus low VAT/SAT and 90-day survival. We next examined the effects of statin therapy and PCSK9 loss-of-function genotype on survival.

**Results:**

Patients with a low VAT/SAT had increased 90-day survival and were relatively protected against low LDL levels in sepsis compared to high VAT/SAT. Statin treatment abrogated the beneficial effects of low VAT/SAT; eliminating the difference in LDL levels and survival between patients with low and high VAT/SAT. PSCK9 loss-of-function genotype similarly eliminated the increased LDL levels in low VAT/SAT patients but, in contrast, increased the survival advantage of low VAT/SAT compared to high VAT/SAT.

**Conclusions:**

Low LDL levels per se are not simply associated with decreased sepsis survival because lowering LDL levels by inhibiting LDL production (statin treatment) is associated with adverse outcomes, while increased LDL clearance (PCSK9 loss-of-function genotype) is associated with improved outcomes in patients with low VAT/SAT.

**Electronic supplementary material:**

The online version of this article (10.1186/s13054-018-1985-1) contains supplementary material, which is available to authorized users.

## Background

A number of studies report that obesity, as indicated by high body mass index (BMI), is paradoxically associated with improved survival during sepsis [1] although this association has not been uniformly observed [2, 3]. Discrepancies between studies may be partly explained by the recent discovery that patients with a high ratio of abdominal visceral adipose tissue to subcutaneous adipose tissue (VAT/SAT) have increased mortality from sepsis compared to patients with low VAT/SAT [4] at any level of BMI [4, 5]. Thus, the distribution of adipose tissue in obesity appears important in sepsis. The mechanism of this VAT/SAT effect is not understood.

We postulated that lipoproteins may be involved since relationships between adipose tissue quantity and lipoprotein levels have been reported in non-septic states [6]. Low LDL levels are a risk factor for sepsis survival and also a risk factor for incidence of sepsis [7]. Since pathogen lipids are sequestered within lipoproteins such as LDL during sepsis, decreased production of LDL leading to low LDL levels may decrease the buffering capacity of LDL for pathogen lipids [8, 9] and allow unbound pathogen lipid to trigger a greater inflammatory response leading to adverse clinical outcomes. In contrast, increased clearance of LDL during sepsis (for example, by loss-of-function genotypes of PCSK9) may be beneficial [10, 11] because pathogen lipids within LDL particles are cleared along with LDL more rapidly [10]. Thus, considering only static serum LDL levels may be too simplistic. Instead, we suggest that it may be necessary to consider the underlying balance of LDL production versus LDL clearance mechanisms when considering prognostic prediction of survival according to LDL levels in sepsis.

To understand potential mechanisms that explain why increased VAT/SAT ratio is harmful in sepsis, we compared two known pathways affecting LDL production and LDL clearance. Statin therapy lowers LDL in the circulation primarily by inhibition of LDL production in hepatocytes [12–14]. Statin treatment is common and therefore sufficiently prevalent in patients presenting with sepsis to hospital emergency departments to serve as a test of the effect of decreased LDL production on LDL levels and survival in sepsis. PCSK9 inhibitors, which increase LDL clearance, have just been approved to treat certain types of hyperlipidemia [15] but their use is not yet sufficiently prevalent to examine their effect on increased LDL clearance in sepsis. We therefore chose the alternative strategy of measuring PCSK9 loss-of-function genotype, which is known to increase LDL clearance. Thus, in patients with low versus high VAT/SAT ratios, we examined the effect of statins to understand the effect of decreased LDL production, and examined the effect of PCSK9 loss-of-function genotype to understand the effect of increased LDL clearance in patients with sepsis.

## Methods

### Patients

We conducted a retrospective analysis of a prospectively accrued cohort of patients with sepsis enrolled from January 2011 to July 2013 at St. Paul’s Hospital, Vancouver, Canada. Patients with suspected sepsis were identified when the Emergency Department physician activated the Institutional Severe Sepsis Order Set. As these patients were recruited prior to the new Sepsis-3 guidelines [16], we abided by the previous sepsis definition, which required patients to have two or more systematic inflammatory response syndrome (SIRS) criteria and have known or suspected infection [17]. Of these patients, we selected those who had an abdominal computed tomography (CT) scan for clinical reasons within 2 months prior to ICU admission, during the hospital stay, or within 1 month after discharge. Statin use and intensity regimen (low, moderate or intense) were determined at the time of study inclusion according to the American College of Cardiology guidelines for statin therapy [18]. All patients provided written informed consent. This protocol was reviewed and approved by the St. Paul’s Hospital/University of British Columbia research ethics board.

### Measurement of adipose tissue area

We measured the visceral adipose tissue area (VAT) and subcutaneous adipose tissue area (SAT) from the abdominal CT scans at three levels: between L2 and L3, L3 and L4, and L4 and L5 as previously reported [4]. All CT scan images were de-identified and then loaded into 3D Slicer (http://slicer.org), an open source software package for medical image visualization and analysis. VAT was identified by manually tracing a region of interest beneath the abdominal wall on the cross-sectional CT scan and adipose tissue area within this region of interest was measured by determining the number of pixels within a window width of − 190 to − 30 Hounsfield units [19–21]. Similarly, SAT was identified by manually tracing the skin surface and abdominal wall on the cross-sectional CT scan and the same Hounsfield unit window width was used to measure SAT. We calculated area and used the average from the three levels ranging from L2 to L5 to reduce noise and errors. This analysis was performed by the same individual for all CT scans while blinded to clinical outcomes.

### LDL measurement

A 6-mL EDTA tube of blood was collected at the time of initial blood culture and immediately placed on ice. Blood was spun at 1800*g for 12 min and total cholesterol (TC), triglyceride (TG) and high-density lipoprotein (HDL)-cholesterol were measured in the plasma fraction on our hospital clinical laboratory ADVIA 1800 Chemistry System (Siemens). LDL measurement was determined using the formula:

$$ \mathrm{LDL}=\mathrm{TC}\hbox{-} \mathrm{HDL}\hbox{-} \mathrm{TG}/5.0. $$

### PCSK9 genotype measurement

Total genomic DNA for genotyping was extracted from the buffy coat fraction using a QIAGEN DNeasy Blood & Tissue Kit (QIAGEN 69506). PCSK9 genotyping was performed in all samples for three common PCSK9 missense loss-of-function variants (minor allele frequency, ≥0.5%): rs11591147 (R46L), rs11583680 (A53V), and rs562556 (I474V) and the gain-of-function variant rs505151 (G670E) using pre-validated TaqMan SNP Genotyping Assays (ThermoScientific 4,351,379). Assays were run on a ViiA7 platform using the system’s existing SNP genotyping software and protocol. All alleles were called using the software clustering algorithm, with a 100% success rate. For quality control, 10% of samples were randomly repeated for each SNP to ensure reproducibility. For simplicity in this genotype analysis we excluded four patients who carried the rare PCSK9 gain-of-function variant.

### Statistical analysis

For all comparisons we divided patients into those with a VAT/SAT below the median value (low VAT/SAT) and those with a VAT/SAT above (high VAT/SAT) [4]. We compared baseline characteristics between low and high VAT/SAT groups using the *t* test for normally distributed variables and the chi square test for proportions. LDL levels were not normally distributed so these data are reported as median and interquartile range, and the Mann-Whitney test was used for comparisons. We tested for differences in 90-day survival curves using the log-rank test. All analyses were conducted in SPSS choosing *p* < 0.05 as significant.

## Results

### Patients

There were 75 patients who fulfilled the inclusion criteria, of which 2 patients did not have an adequate quality CT scan, leaving 73 patients for the measurement of VAT/SAT, LDL, statin use, PCSK9 genotype, and other secondary parameters. The intra-observer reproducibility was high (intraclass correlation coefficient = 0.99) for both VAT and SAT measurements. The median value of the VAT/SAT ratio was 0.80 (IQR 0.50–1.03). There were 36 patients with a calculated VAT/SAT ratio lower than 0.80 and these were classified into the low VAT/SAT group, while 37 patients had a calculated VAT/SAT ratio above 0.80 and were classified into the high VAT/SAT group. The proportion of patients using statins was 22.2% (8/36) in the low VAT/SAT group and was 40.5% (15/37) in the high VAT/SAT groups. PCSK9 LOF genotype was present in 57.1% (20/35) of patients from the low VAT/SAT group and in 44.1% (15/34) of patients from the high VAT/SAT group.

### Baseline characteristics

The baseline characteristics of patients in both low and high VAT/SAT groups were similar except for age, gender, creatinine, and acute physiology and chronic health evaluation II (APACHE II) scores (Table 1). Compared to low VAT/SAT, patients with high VAT/SAT were older (*p* = 0.025), more often male (*p* < 0.001), had higher APACHE II scores (*p* = 0.028), and higher creatinine levels at admission (*p* = 0.039). Type of statins and intensity regimen re described in Additional file 1: Table S1.

**Table 1:** Characteristics of patients at baseline

Variable	Visceral to subcutaneous adipose tissue ratio (VAT/SAT)		
	< median (n = 36)	≥ median (*n* = 37)	*p* value
Baseline characteristics			
Age, (IQR)	38–70	56–68	0.025
Male, *n* (%)	18 (50.0)	35 (94.6)	<0.001
Caucasians, *n* (%)	31 (93.9)^a^	20 (80.0)	0.145
Chronic condition, *n* (%)			
Hypertension	14 (38.9)	12 (32.4)	0.740
Congestive heart failure	4 (11.1)	3 (8.1)	0.711
Myocardial infarction	3 (8.3)	3 (8.1)	0.136
Diabetes mellitus	9 (25.0)	10 (27.0)	1.000
COPD	5 (13.9)	11 (29.7)	0.176
Chronic kidney disease	2 (5.6)	6 (16.2)	0.261
Cirrhosis	1 (2.8)	4 (10.8)	0.358
Malignancy	2 (5.6)	2 (5.4)	1.000
Hemodynamic variables at admission (IQR)			
Mean arterial pressure	66–89	72–84	0.911
Heart rate	83–104	79–115	0.704
Respiratory rate^b^	16–20	17–22	0.323
Temperature (°C)	36.4–37.2	36.3–37.1	0.661
Laboratory measurements at admission (IQR)			
Hemoglobin (g/L)	88.0–130.0	87.0–127.0	0.467
WBC (×10^3^/mm^3^)	5.90–12.97	6.67–17.65	0.220
Platelets (10^9^/L)	128–362	119–230	0.075
Creatinine (mmol/L)	64–119	77–180	0.039
Lactate (mmol/L)	1.1–1.9	1.3–3.3	0.179
Other clinical characteristics			
APACHE II score (IQR)	3–12	8–17	0.028
Use of vasopressor, *n* (%)	6 (16.7)	6 (16.2)	0.871
Mechanical ventilation, *n* (%)	12 (33.3)	14 (37.8)	0.444
PCSK9 genotype, *n* (%)			
One or more loss-of-function	20 (55.6)	15 (40.5)	0.294
Two or more loss-of-function	5 (13.9)	9 (24.3)	0.404
Adipose tissue area (cm^2^) (IQR)			
Visceral adipose tissue	44.7–138.5	126.8–279.5	<0.001
Subcutaneous adipose tissue	116.5–270.8	99.8–212.2	0.300

*Abbreviations: VAT* visceral abdominal tissue, *SAT* subcutaneous adipose tissue, *IQR* interquartile range, *COPD* chronic obstructive pulmonary disease, *WBC* white blood cell, *APACHE II* acute physiology and chronic health evaluation II

^a^Missing data, *n* = 3

^b^Breaths per minute

### Relationship between VAT/SAT and LDL levels

**Fig. 1:**
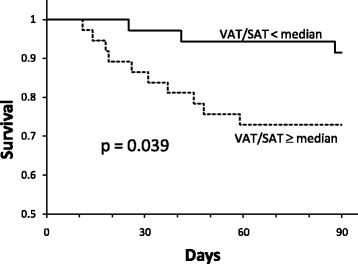
Ninety-day mortality in the low and high visceral adipose tissue/subcutaneous adipose tissue (VAT/SAT) groups. Patients with a VAT/SAT ratio lower than the median value (*n* = 36) had longer survival compared to patients with a VAT/SAT ratio higher than the median value (*n* = 37) (*p* = 0.039). This observation is consistent with a previous report in patients with septic shock [4]

**Fig. 2:**
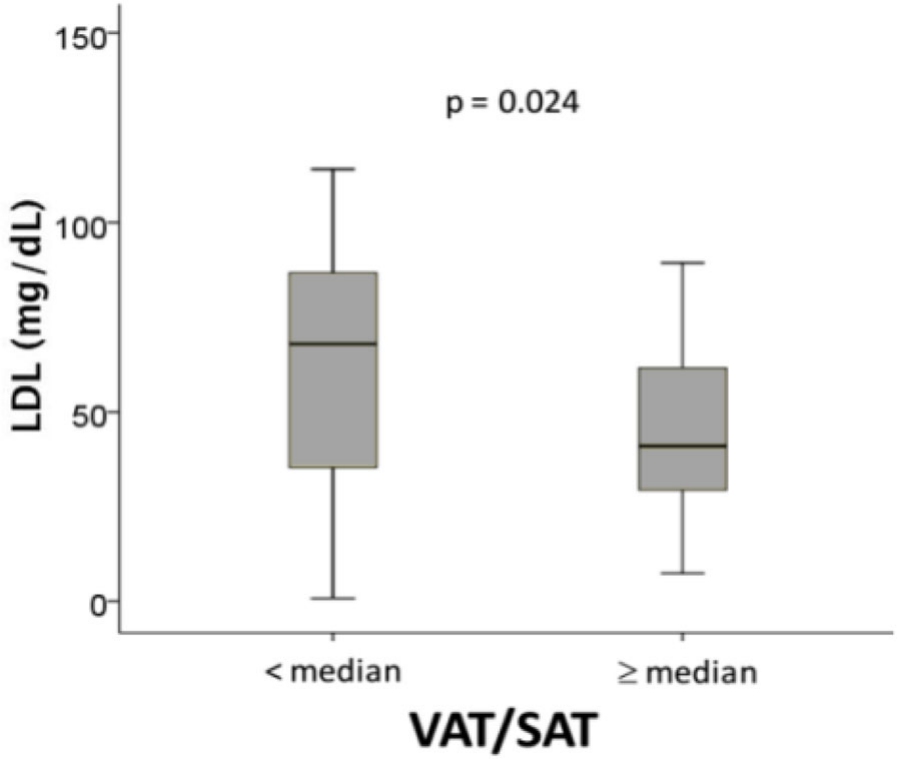
Patients with a visceral adipose tissue/subcutaneous adipose tissue (VAT/SAT) ratio lower than the median value (*n* = 36) had higher low-density lipoprotein (LDL) levels (median and interquartile range, mg/dL) compared to patients with a VAT/SAT ratio greater than the median value (*n* = 37) (*p* = 0.024)

Similar to a previous report in a septic shock cohort [4], patients with a low VAT/SAT ratio (below the median of 0.802) had higher 90-day survival in the current sepsis cohort (*p* = 0.039) (Fig. 1). Patients with a low VAT/SAT ratio had higher LDL compared to patients having a high VAT/SAT (*p* = 0.024) (Fig. 2).

### Effect of statins on LDL levels according to VAT/SAT group

**Fig. 3:**
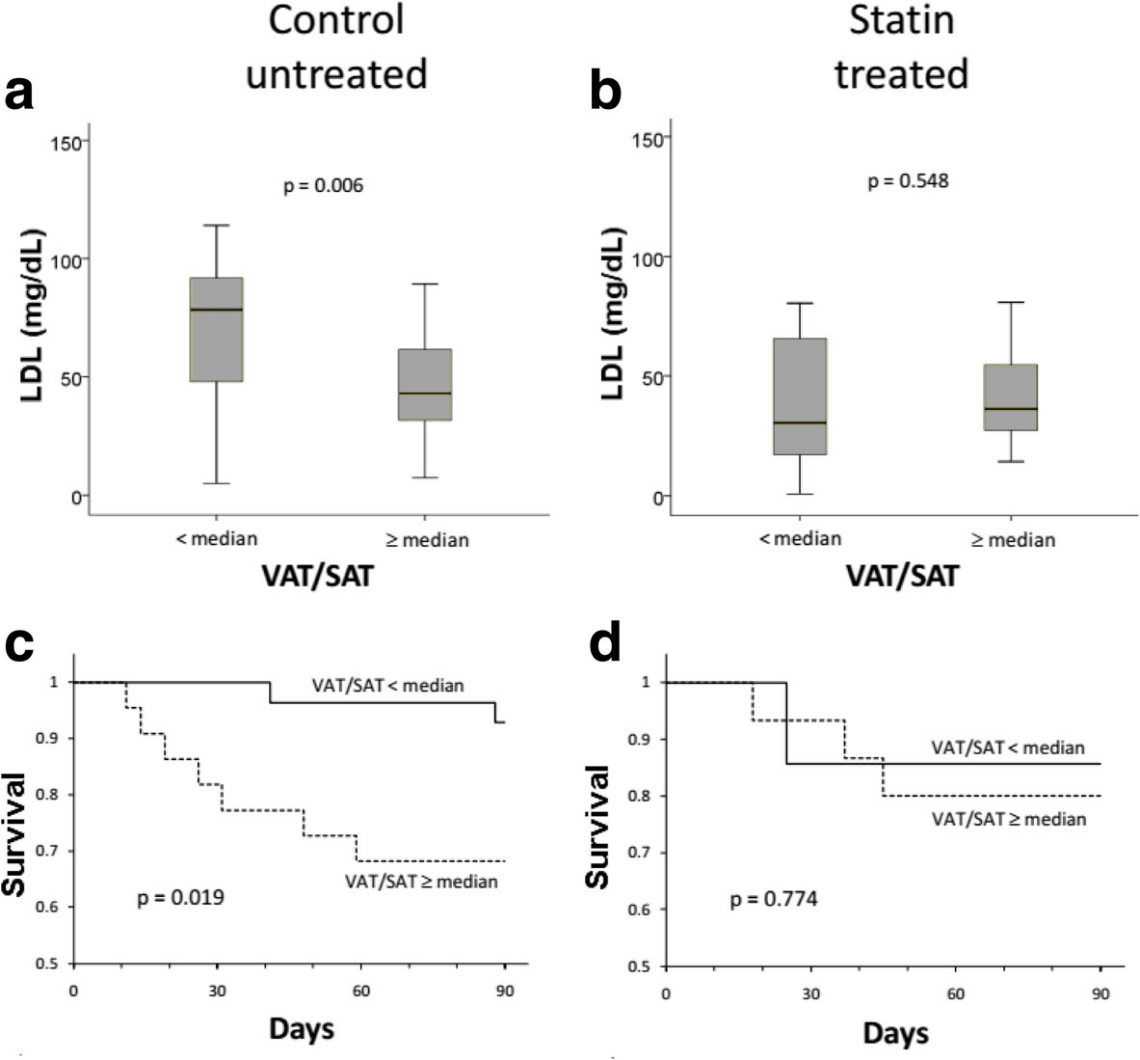
**a** Untreated control patients not on statins (*n* = 50) with low visceral adipose tissue/subcutaneous adipose tissue (VAT/SAT) (*n* = 28) have higher low-density lipoprotein (LDL) (median and interquartile range, mg/dL) than patients with high VAT/SAT (*n* = 22) (*p* = 0.006). **b** Statin-treated patients (*n* = 23) have low LDL regardless of VAT/SAT category (*p* value not significant). **c** Control untreated patients not on statins with low VAT/SAT (*n* = 28) have longer survival compared to patients with high VAT/SAT (*n* = 22) (*p* = 0.019). **d** In statin-treated patients (*n* = 23) there is no difference in survival between VAT/SAT categories (*p* value not significant)

The beneficial effect of low VAT/SAT was most pronounced in control patients not treated with statins (Fig. 3a). Patients with low VAT/SAT not on statin therapy had LDL levels that were significantly higher than those with high VAT/SAT not on statin therapy (*p* = 0.006) (Fig. 3a). Thus, low VAT/SAT protected against the very low LDL levels observed in patients with sepsis. In these patients the higher LDL levels in the low VAT/SAT group were associated with significantly higher 90-day survival (*p* = 0.019) (Fig. 3c).

Statin treatment abrogated these beneficial effects. Statin treatment reduced LDL in patients with low VAT/SAT so that patients with low or high VAT/SAT had similarly low LDL levels (*p* = 0.548) (Fig. 3b). Loss of the difference in LDL levels in patients with low versus high VAT/SAT was also associated with loss of the difference in 90-day survival (*p* = 0.774) (Fig. 3d). Thus, statin treatment reveals that decreased LDL production leads to loss of the LDL-preserving and survival benefit of low VAT/SAT versus high VAT/SAT.

### Effect of PCSK9 loss-of-function genotype

**Fig. 4:**
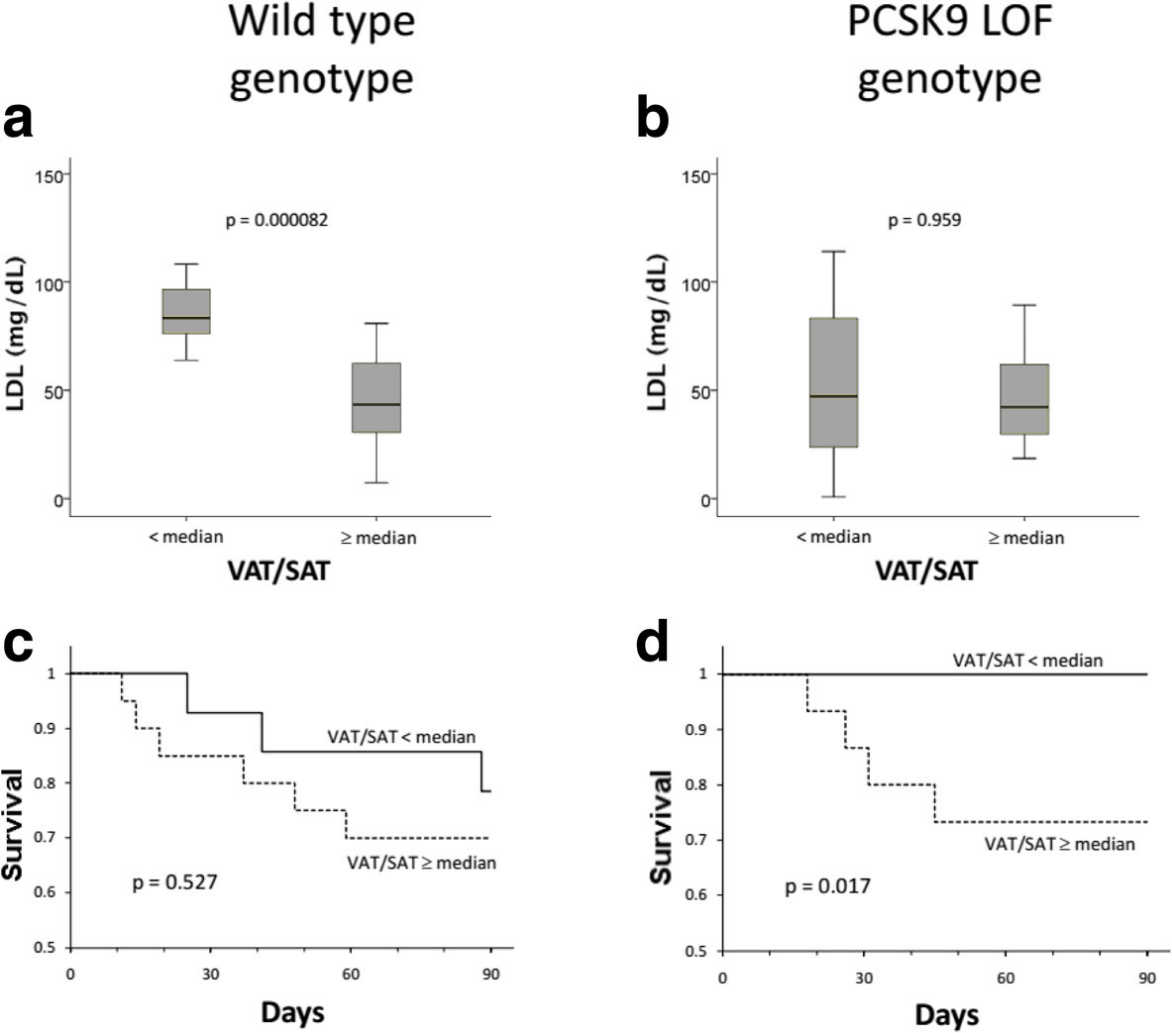
**a** Patients who have the wild type genotype (*n* = 34) with low visceral adipose tissue/subcutaneous adipose tissue (VAT/SAT) (*n* = 15) have higher low-density lipoprotein (LDL) (median and interquartile range, mg/dL) than patients with high VAT/SAT (*n* = 19) (*p* = 0.000082). **b** Patients who have PCSK9 loss-of-function genotype (*n* = 35) have low LDL levels regardless of VAT/SAT category (*p* value not significant). **c** In patients who have wild type genotype (*n* = 34) there is no significant difference in survival between those with low VAT/SAT (*n* = 15) or high VAT/SAT (*n* = 19) (*p* value not significant). **d** Patients who have PCSK9 loss-of-function genotype (*n* = 35) with low VAT/SAT (*n* = 20) have longer survival compared to patients with high VAT/SAT (*n* = 15) (*p* = 0.017). Four patients who carried PCSK9 gain-of-function mutations were excluded from this analysis (*n* = 69)

Similar to patients not taking statins, patients with PCSK9 wildtype genotype in the low VAT/SAT group had preserved LDL levels that were significantly higher than those in patients with high VAT/SAT (*p* < 0.001) (Fig. 4a). Similar to statin treatment, PCSK9 loss-of-function genotype in the patients with low VAT/SAT abrogated the effect of VAT/SAT on LDL such that patients with low or high VAT/SAT had similarly low LDL levels (*p* = 0.959) (Fig. 4b).

In distinct contrast to statin-treated patients, loss of the difference in LDL levels by PCSK9 genotype in patients with low versus high VAT/SAT was not associated with loss of the survival benefit. In fact, PCSK9 loss-of-function genotype was associated with greater difference between improved survival in patients with low VAT/SAT compared to all patients with high VAT/SAT (*p* = 0.017 and *p* = 0.527) (Fig. 4d versus Fig. 4c). Thus, PCSK9 loss-of-function genotype demonstrates that low LDL per se does not lead to increased mortality because low LDL levels in the PCSK9 loss-of-function genotype patients with low VAT/SAT was associated with remarkably high survival. These analyses were performed according to cholesterol levels at sepsis admission and demonstrated similar results in comparison to LDL analysis (Additional file 1: Figures S1 to S3).

### Effect of statins and PCSK9 loss-of-function genotype on LDL levels in the low VAT/SAT group

In order to rule out a possible beneficial effect of statin use on survival, and to confirm our hypothesis that LDL clearance (and not decreased synthesis) in sepsis confers a survival benefit, we performed a subgroup analysis only in patients with low VAT/SAT ratio. In these patients, both statin-treated and PCSK9 LOF genotype groups had significantly lower LDL compared to the control and wild type (WT) groups, respectively (*p* = 0.046 and *p* = 0.028) (Additional file 1: Figures S4A and S4B). However, despite this observation, only the PCSK9 LOF group yielded a significant mortality benefit compared to WT, whereas no appreciable differences in survival were observed between statin treated and untreated control groups (Additional file 1: Figure S5A and S5B).

### Effect of statins on survival in the high VAT/SAT group

In patients with high VAT/SAT, statin treatment did not demonstrate a survival benefit compared to untreated control groups (Additional file 1: Figure S6).

## Discussion

Both statin treatment and PCSK9 loss-of-function took away the LDL-preserving effect of low VAT/SAT resulting in uniformly low LDL across both low and high VAT/SAT groups. Despite this similarity in LDL levels, the survival rates were not alike. When LDL was lowered using statins the survival benefit conferred from having a low VAT/SAT was eliminated. In contrast, PCSK9 loss-of-function genotype-driven changes in LDL levels (by increasing LDL clearance) were associated with statistically significantly improved outcomes. In other words, while PCSK9 loss-of-function led to the low VAT/SAT group losing their ability to preserve LDL levels, it did not take away the protective effect, and in fact, enhanced the survival benefit. Thus, it is not the static low LDL levels in sepsis that drive increased mortality. Rather, it may be the dynamic interplay between LDL production and clearance that contributes to clinical outcomes in sepsis.

These VAT/SAT observations in an independent cohort replicate the finding by Pisitsak et al. that low VAT/SAT is associated with longer survival in sepsis compared to high VAT/SAT [4]. Patients with sepsis in the current study were identified earlier in sepsis in the Emergency Department rather than in the ICU and therefore had less severe illness and lower mortality compared to the patients with septic shock who were recruited from an ICU, as described in the previous report [4]. In addition to this, we also found that the low VAT/SAT group was able to preserve their LDL levels at baseline (considering no statin treatment and PCSK9 wildtype genotype patients). This preservation of LDL levels was associated with increased survival. This observation is also concordant with previous studies suggesting that low LDL is associated with worse outcomes [7]. However, these previous studies did not examine the effect of the underlying determinants of LDL levels – LDL production (as altered by statins) and LDL clearance (as altered according to PCSK9 genotype). Statin therapy lowers LDL in the circulation primarily via the inhibition of LDL production in hepatocytes and, to a lesser extent, via a resulting feedback increase in LDL uptake by upregulation of LDL receptor expression (12). That statins primarily inhibit LDL production is confirmed by the observation that LDL levels are decreased by statins in patients with homozygous familial hypercholesterolemia, where LDL receptors are not functioning [13, 14].

LDL serves an important role in buffering the response to endotoxins by forming an endotoxin-lipoprotein complex [22, 23]. In vitro studies have demonstrated that lipopolysaccharide (LPS) binds readily to serum lipoproteins, and that LPS-lipoprotein complexes are less toxic than unbound LPS [24, 25]. This LPS-lipoprotein-complex formation offers protection in an acute setting, but may also be implicated in the pathogenesis of atherosclerosis [26]. In either scenario, there is value to increasing the clearance of toxin-bound lipoproteins because they may otherwise increase the inflammatory response, leading to unfavorable clinical outcomes in sepsis and/or contribute to atherogenesis. In major inflammatory states, there is a reduction in plasma levels of LDL in parallel with other lipoproteins [27]. The mechanism behind our observation of patients with low VAT/SAT having the ability to preserve their LDL levels (no statin treatment and PCSK9 wildtype genotype) compared to those with high VAT/SAT is yet to be fully understood.

Although static LDL levels have some value for estimating the buffering capacity of the patient against endotoxins in sepsis, it does not fully explain the complex dynamic role of LDL production and clearance, which interact to determine plasma LDL levels. Our results show that there are marked differences between modulating LDL production and LDL clearance in understanding the association between LDL levels and survival in sepsis. Thus, it may be incomplete and in some cases incorrect to conclude that absolute LDL levels are of primary importance in sepsis. Rather, decreased production of LDL may contribute to adverse outcomes but increased clearance appears to be associated with beneficial outcomes. Perhaps this is because inhibiting the production of LDL hinders the buffering capacity of the body against LPS, whereas increased clearance helps eliminate the LDL-endotoxin complex.

Our results further highlight the differential contributions from VAT and SAT in sepsis, which cannot be explained by BMI alone. Several factors that contribute differently towards VAT and SAT have been established, including gender, age, and the onset of menopause in females [28]. When looking at VAT/SAT ratios, one study suggested that VAT/SAT ratios are closely associated with age [5], while another found the opposite when looking at young Japanese male subjects [29]. As such, our cohort showed great age variability on analysis according to low and high VAT/SAT ratios. In parallel with its inflammatory properties, adipose tissue is implicated in the development of metabolic conditions, so adipose tissue is both an active immune and endocrine organ [30]. VAT is a known predictor of diabetes mellitus, while SAT is not [31]. An analysis of the Framingham Heart Study found that high VAT/SAT ratio was associated with increased cardiometabolic risk [5]. Our results identify a novel difference between VAT and SAT by modulating lipoprotein levels differentially during sepsis. Furthermore, both statins [32] and PCSK9 [33] may also be differentially related to visceral versus subcutaneous obesity. In health, LDL and other lipoprotein particles are correlated more strongly with visceral adipose tissue (VAT) than with subcutaneous adipose tissue (SAT) [34].

Statin use may not improve or worsen survival outcomes on a uniform basis in sepsis and our findings may help elucidate the discrepancy found in currently available statin versus sepsis survival studies [35, 36]. A meta-analysis by Wan et al. showed that prospective studies found no benefit of statin use in sepsis survival, while retrospective studies have demonstrated statin benefit [36]. One prospective double-blind, randomized, controlled trial in 2012 (ASEPSIS trial) found that acute administration of atorvastatin was associated with less progression to severe sepsis, but did not change survival [37]. In addition, Beed et al. showed that prior statin use had no benefit in patients who developed sepsis [38], while other studies report benefit of statins on sepsis survival [39, 40]. Our results demonstrate that VAT/SAT status – and other factors – may influence the effect of statins on survival in sepsis.

Limitations of our study include but are not limited to the inherent biases of a retrospective study design and that the design is an association study so causal inference is not possible. Because our study started before publication of the new Sepsis-3 [16] definition we used the previous definition of sepsis in patients at first contact in the Emergency Department. This means that our patients had a wide range of severity of illness and included a significant contribution from patients without organ failure who had low mortality rates. A weakness of this is that our patient population is more heterogeneous than if the Sepsis-3 definition were used. A strength of this is that our findings may be more broadly applicable. Patients in our study only had abdominal CT scans when the treating physician ordered them for clinical purposes, and this may present an unknown bias. We used PCSK9 loss-of-function genotype as a surrogate to mark PCSK9 inhibition in vivo; however, it is unclear whether our PCSK9 genotype findings will translate to effects on sepsis outcomes by modulating PCSK9 with PCSK9-inhibiting drugs. We took into account the production and clearance of LDL in lowering LDL, but there may be other important mechanisms involved in LDL homeostasis (e.g. sequestration by macrophages). Further studies looking into this these VAT/SAT subgroups and their immunogenic response, or lack thereof, to statin therapy and the implications of PCSK9 loss-of-function genotype will be necessary to elucidate causal pathways.

In conclusion, low VAT/SAT is associated with relative preservation of LDL levels during sepsis and improved survival. However, low LDL levels per se do not appear to cause decreased sepsis survival because inhibiting LDL production with statins (which lowers LDL levels) is associated with adverse outcomes, while lowering plasma LDL by increasing LDL clearance (PCSK9 loss-of-function genotype) was associated with higher survival in patients with a low VAT/SAT.

## Conclusions

Low LDL levels per se are not simply associated with decreased sepsis survival, because lowering LDL levels by inhibiting LDL production (statin treatment) is associated with adverse outcomes, while increased LDL clearance (PCSK9 loss-of-function genotype) is associated with improved outcomes in patients with low VAT/SAT. Increased LDL clearance during sepsis may be a useful therapeutic goal.

## Additional file

Additional file 1: **Table S1.** Statin subtypes and dosages. **Figure S1.** Cholesterol levels according to VAT/SAT groups. Higher cholesterol levels were observed in the low VAT/SAT group (*n* = 36) than high VAT/SAT group (*n* = 37) (*p* = 0.005). **Figure S2A.** Cholesterol levels in untreated control patients according to VAT/SAT groups. In control untreated patients (*n* = 50), low VAT/SAT group (*n* = 28) had higher cholesterol levels than high VAT/SAT group (*n* = 22) (*p* = 0.009). **Figure S2B.** Cholesterol levels in statin treated patients according to VAT/SAT groups. Patients on statin treatment (*n* = 23) showed no difference in cholesterol levels between low (*n* = 8) and high VAT/SAT (*n* = 15) groups (*p* value not significant). **Figure S3A.** Cholesterol levels in WT genotype patients according to VAT/SAT groups. Patients with WT genotype (*n* = 34) had higher cholesterol levels in the low VAT/SAT group (*n* = 15) compared to the high VAT/SAT group (*n* = 19) (*p* = 0.001). **Figure S3B.** Cholesterol levels in patients with the PCSK9 LOF genotype according to VAT/SAT group. No differences in cholesterol levels were observed between the low (*n* = 20) and high VAT/SAT (*n* = 15) group in patients with PCSK9 LOF (*n* = 35) (*p* value not significant). **Figure S4A.** Patients with low VAT/SAT (*n* = 36) who have been treated with statins (*n* = 8) have lower LDL levels than untreated control patients (*n* = 28) (*p* = 0.046). **Figure S4B.** Patients with low VAT/SAT with PCSK9 LOF genotype (n = 20) have lower LDL levels than WT patients (*n* = 15) (*p* = 0.028). **Figure S5A.** In patients with low VAT/SAT, there is no significant difference in survival between the untreated control (*n* = 28) and statin-treated groups (*n* = 8) (*p* = 0.485). **Figure S5B.** Patients with low VAT/SAT who have the PCSK9 LOF genotype (*n* = 20) have increased survival compared to patients with WT (*n* = 15) (*p* = 0.043). **Figure S6.** In patients with high VAT/SAT (*n* = 37), statin treatment did not demonstrate a survival benefit compared to the untreated control groups (*p* = 0.410). (DOCX 641 kb)
