# Supplementary material for: Survival benefit of a low ratio of visceral to subcutaneous adipose tissue depends on LDL clearance versus production in sepsis
Source: Crit Care. 2018 Mar 6;22:58. doi: 10.1186/s13054-018-1985-1 (PMC5840798; doi:10.1186/s13054-018-1985-1)
Supplement: Supplementary file 1 — Table S1. Statin subtypes and dosages. Figure S1. Cholesterol levels according to VAT/SAT groups. Higher cholesterol levels were observed in the low VAT/SAT group (n = 36) than high VAT/SAT group (n = 37) (p = 0.005). Figure S2A. Cholesterol levels in untreated control patients according to VAT/SAT groups. In control untreated patients (n = 50), low VAT/SAT group (n = 28) had higher cholesterol levels than high VAT/SAT group (n = 22) (p = 0.009). Figure S2B. Cholesterol levels in statin treated patients according to VAT/SAT groups. Patients on statin treatment (n = 23) showed no difference in cholesterol levels between low (n = 8) and high VAT/SAT (n = 15) groups (p value not significant). Figure S3A. Cholesterol levels in WT genotype patients according to VAT/SAT groups. Patients with WT genotype (n = 34) had higher cholesterol levels in the low VAT/SAT group (n = 15) compared to the high VAT/SAT group (n = 19) (p = 0.001). Figure S3B. Cholesterol levels in patients with the PCSK9 LOF genotype according to VAT/SAT group. No differences in cholesterol levels were observed between the low (n = 20) and high VAT/SAT (n = 15) group in patients with PCSK9 LOF (n = 35) (p value not significant). Figure S4A. Patients with low VAT/SAT (n = 36) who have been treated with statins (n = 8) have lower LDL levels than untreated control patients (n = 28) (p = 0.046). Figure S4B. Patients with low VAT/SAT with PCSK9 LOF genotype (n = 20) have lower LDL levels than WT patients (n = 15) (p = 0.028). Figure S5A. In patients with low VAT/SAT, there is no significant difference in survival between the untreated control (n = 28) and statin-treated groups (n = 8) (p = 0.485). Figure S5B. Patients with low VAT/SAT who have the PCSK9 LOF genotype (n = 20) have increased survival compared to patients with WT (n = 15) (p = 0.043). Figure S6. In patients with high VAT/SAT (n = 37), statin treatment did not demonstrate a survival benefit compared to the untreated control groups (p = 0.410). (DO [file 13054_2018_1985_MOESM1_ESM.docx]

**Survival benefit of a low ratio of visceral to subcutaneous adipose tissue depends on LDL clearance versus production in sepsis**

**Supplemental Digital Content**

Joseph G. H. Lee^1^, Kelly R. Genga^1^, Chawika Pisitsak^1,2^, John H. Boyd^1^,

Alex K. K. Leung^1^, James A. Russell^1^, Keith R. Walley^1^

^1^Centre for Heart Lung Innovation, University of British Columbia, Vancouver, BC, Canada

^2^Ramathibodi Hospital, Faculty of Medicine, Mahidol University, Bangkok, Thailand

Correspondence:

Keith R. Walley, MD, Centre for Heart Lung Innovation, St. Paul's Hospital, 1081 Burrard Street. Vancouver, BC, Canada V6Z 1Y6

Phone: (604) 806-8136; Email: Keith.Walley@hli.ubc.ca

**Table S1. Statin Subtypes and Dosages**

| Statin | Dose | N | Intensity | 90-day mortality (N) |
| --- | --- | --- | --- | --- |
| Atorvastatin | 20mg | 5 | M | 0 |
| Atorvastatin | 40mg | 4 | H | 3 |
| Atorvastatin | 80mg | 3 | H | 1 |
| Pravastatin | 60mg | 1 | M | 0 |
| Rosuvastatin | 10mg | 2 | M | 0 |
| Simvastatin | 20mg | 2 | M | 0 |
| Simvastatin | 40mg | 6 | M | 1 |

*All patients were re-ordered the same type of statin they were taking prior to hospitalization while admitted.

**Only one patient was ordered a lower dose of statin (Atorvastatin 40mg) than their home dose (Atorvastatin 80mg); otherwise, all others continued their home dose.

Abbreviations: M=moderate; H=high


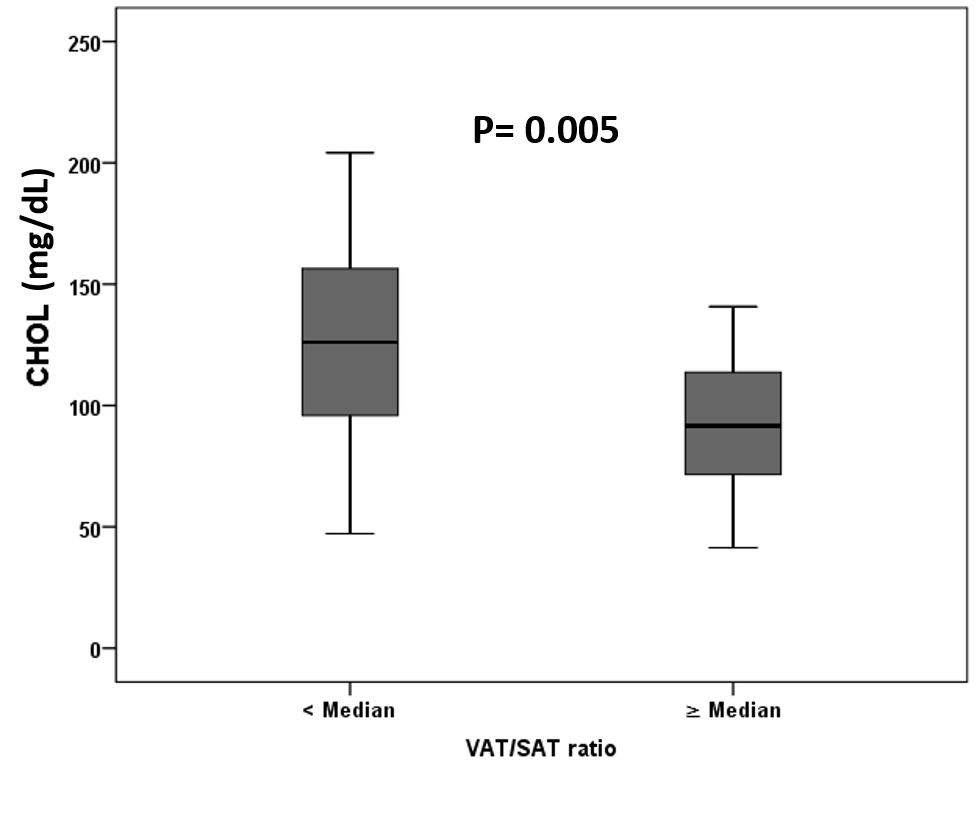
**Figure S1.** Cholesterol levels according to VAT/SAT groups. Higher cholesterol levels were observed in the low VAT/SAT group (n=36) than high VAT/SAT group (n=37) (p=0.005).


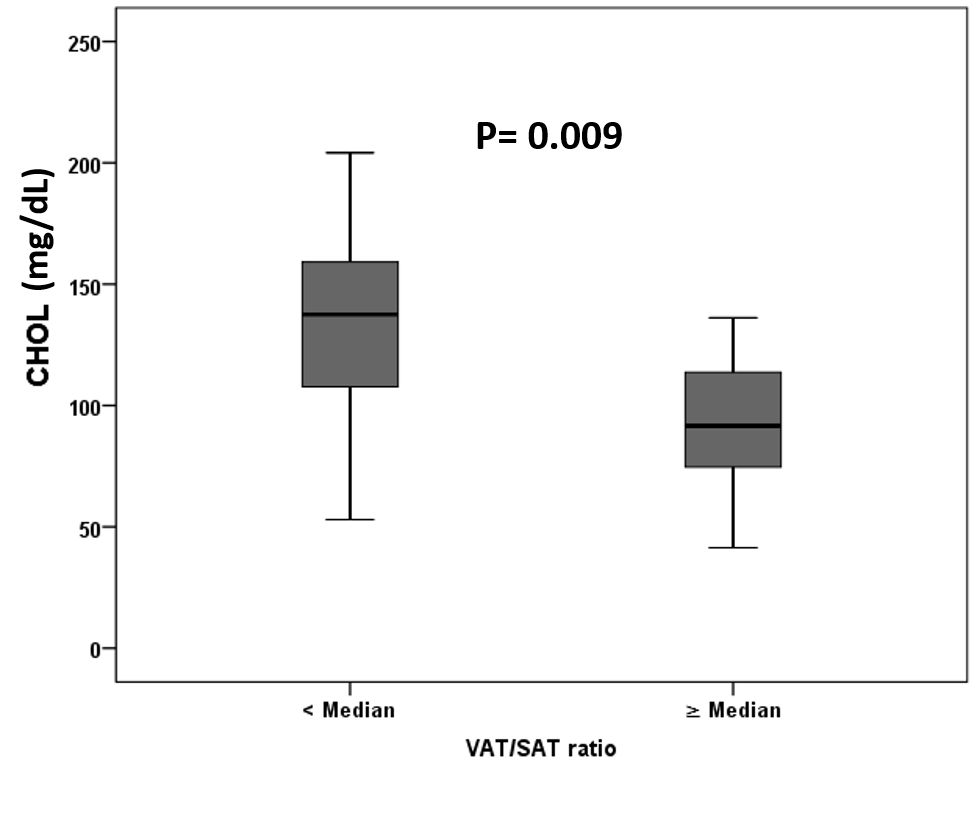


**Figure S2A.** Cholesterol levels in Control Untreated patients according to VAT/SAT groups.

In control untreated patients (n=50), low VAT/SAT group (n=28) had higher cholesterol levels than high VAT/SAT group (n=22) (p=0.009).


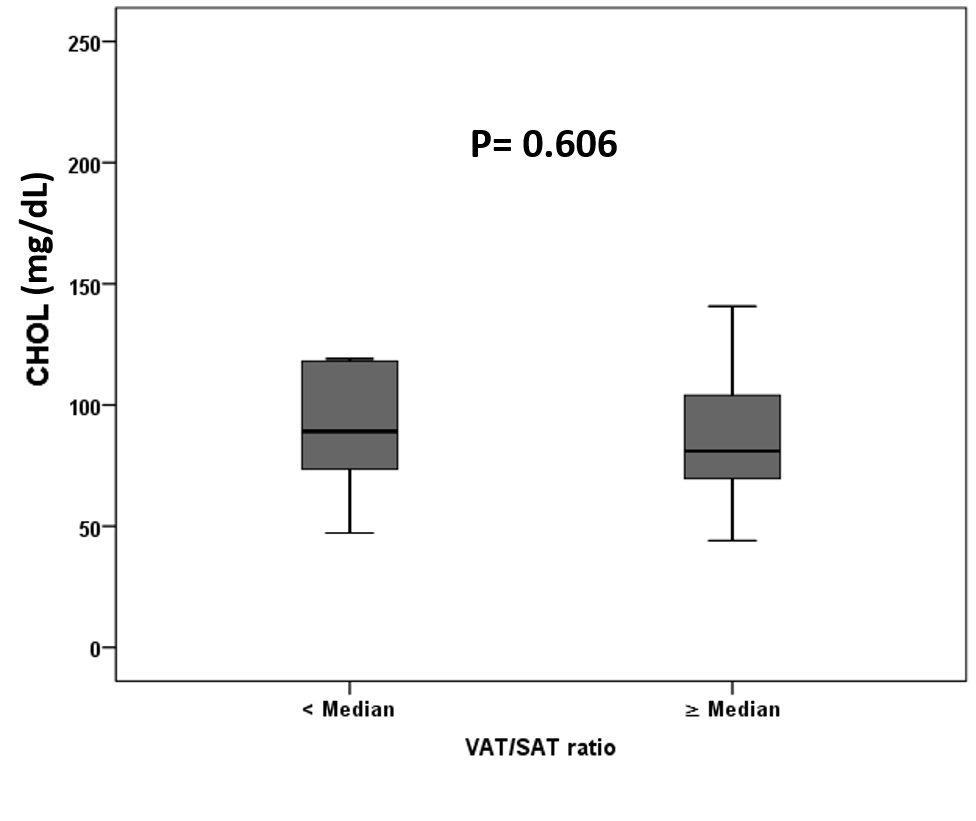


**Figure S2B.** Cholesterol levels in Statin Treated patients according to VAT/SAT groups.

Patients on statin treatment (n=23) showed no difference in cholesterol levels between low (n=8) and high VAT/SAT (n=15) groups (p=NS).


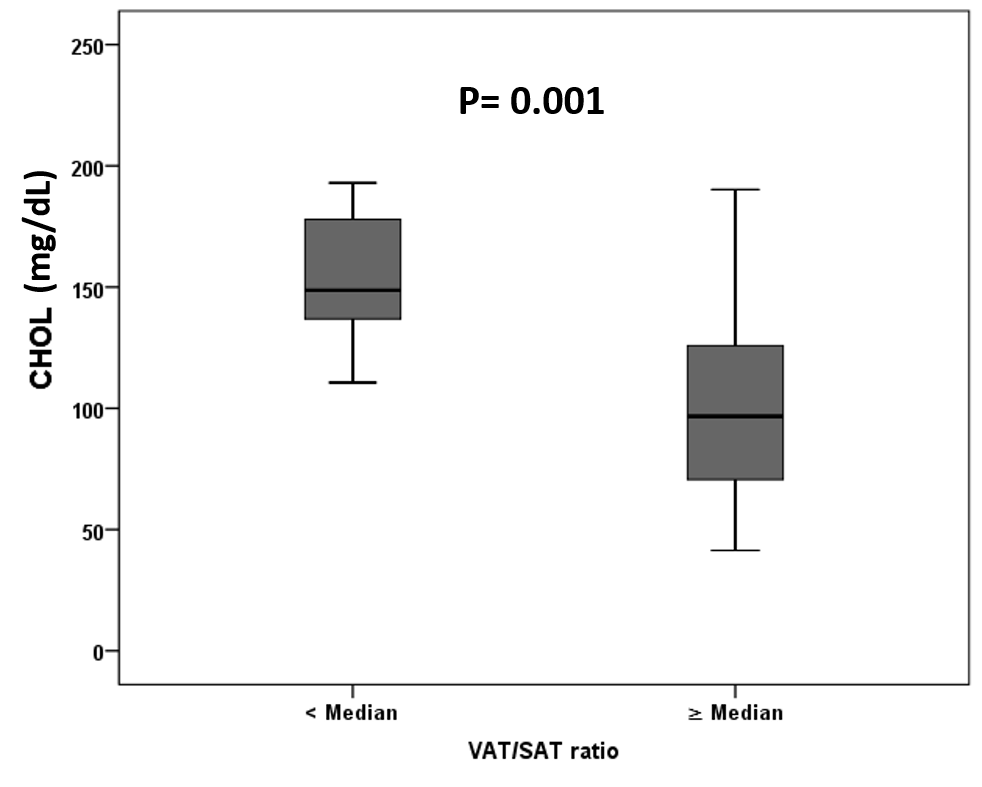


**Figure S3A.** Cholesterol levels in WT genotype patients according to VAT/SAT groups.

Patients with WT genotype (n=34) showed higher cholesterol levels in the low VAT/SAT group (n=15) compared to high VAT/SAT group (n=19) (p=0.001).


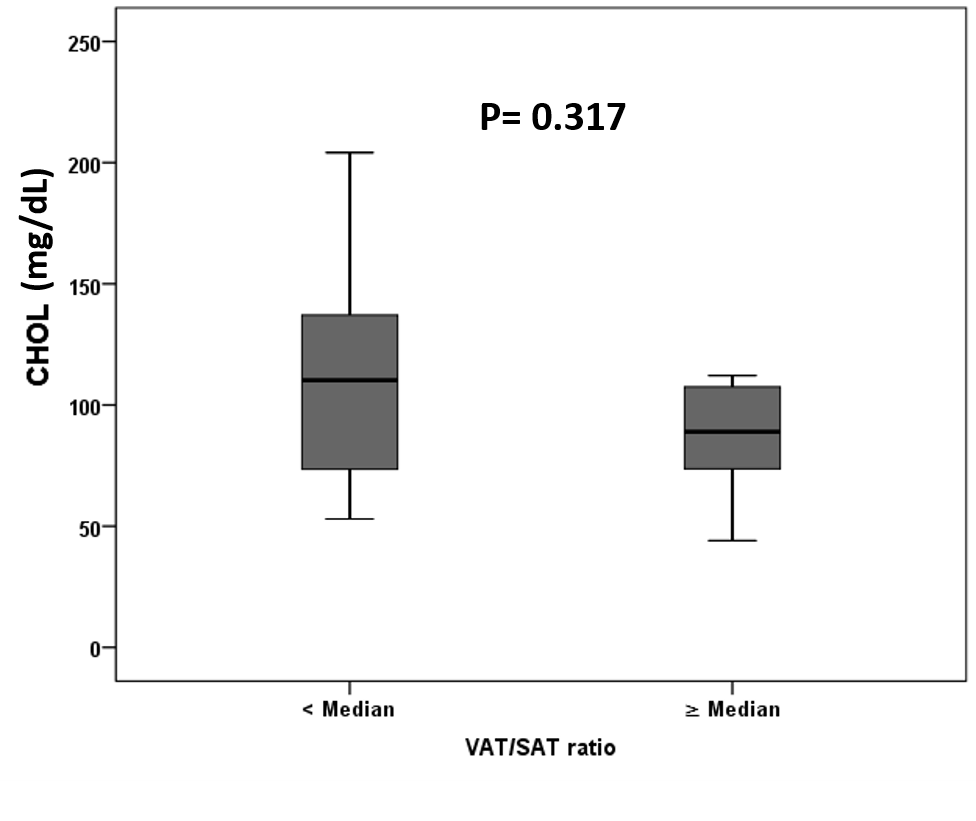


**Figure S3B.** Cholesterol levels in PCSK9 LOF genotype patients according to VAT/SAT group.

No differences in cholesterol levels were observed between low (n=20) and high VAT/SAT (n=15) group in patients with PCSK9 LOF (n=35) (p=NS).


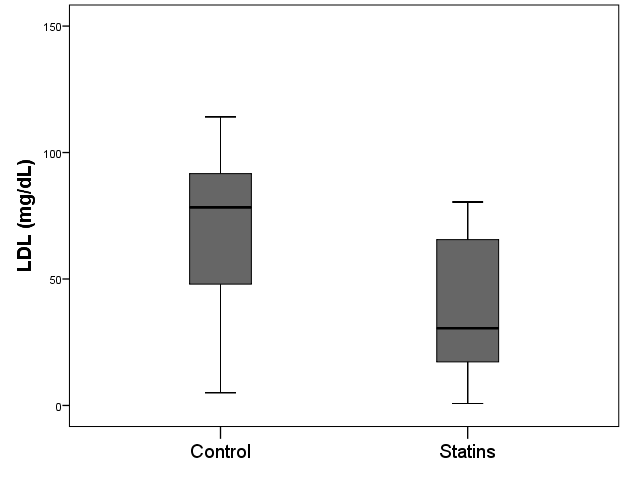


**P= 0.046**

**Figure S4A.** Patients with low VAT/SAT (n=36) who have been treated with statins (n=8) have lower LDL levels than control untreated patients (n=28) (p=0.046).


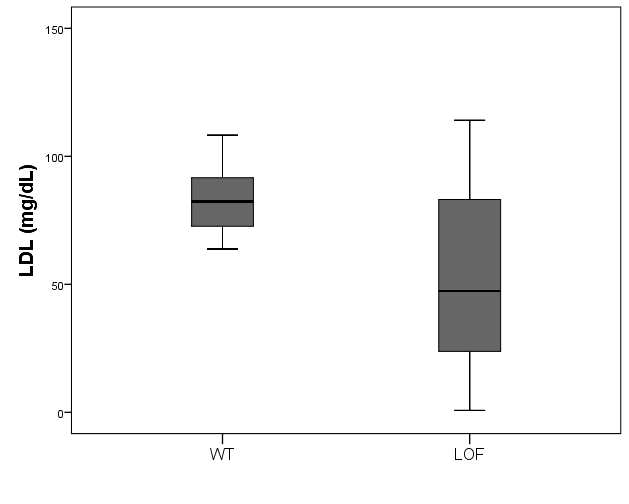


**P= 0.028**

**Figure S4B.** Patients with low VAT/SAT with PCSK9 LOF genotype (n=20) have lower LDL levels than WT patients (n=15) (p=0.028).


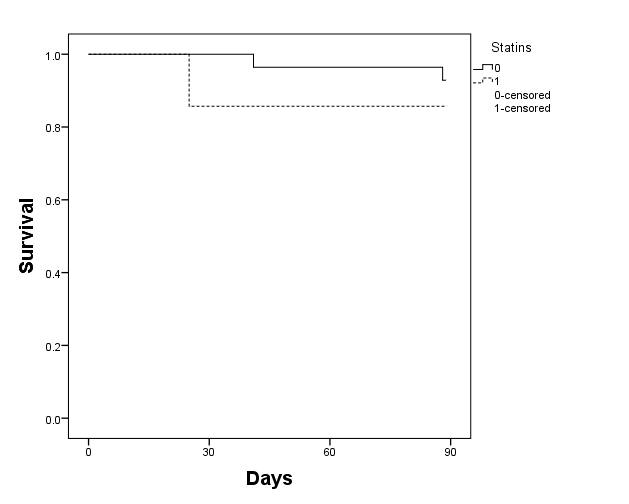


Controls

Statins

P=0.485

**Figure S5A.** In patients with low VAT/SAT, there is no significant difference in survival between control untreated (n=28) and statin treated groups (n=8) (p=0.485).


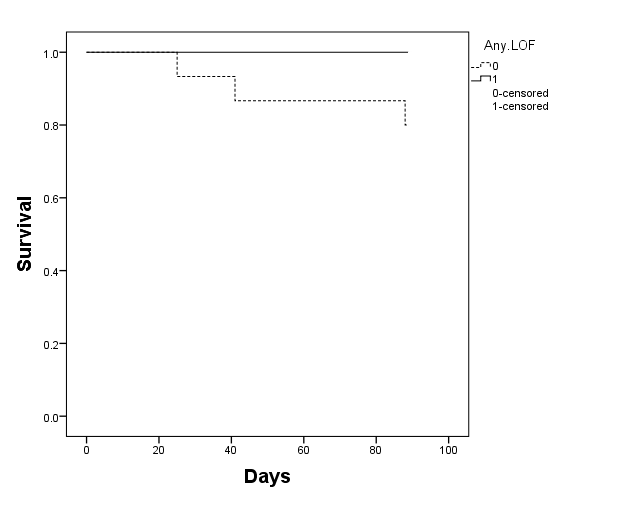


P=0.043

WT

LOF

**Figure S5B.** Patients with low VAT/SAT who have the PCSK9 LOF genotype (n=20) have increased survival compared to WT (n=15) (p=0.043).


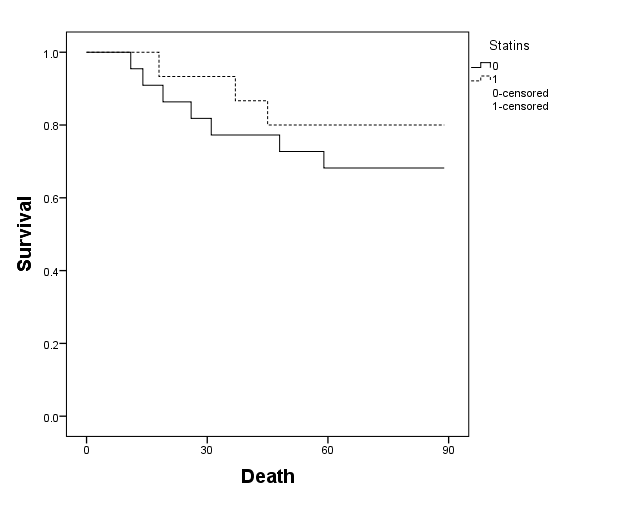


P=0.410

Controls

Statins


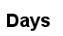


**Figure S6.** In patients with high VAT/FAT (n=37), statin treatment did not demonstrate a survival benefit compared to untreated control groups (p=0.410).
